# CT imaging analysis differentiating papillary renal neoplasm with reverse polarity from papillary renal cell carcinoma: combined with a radiomics model

**DOI:** 10.1007/s11604-024-01631-2

**Published:** 2024-07-24

**Authors:** Hyo Jeong Lee, Taek Min Kim, Jeong Yeon Cho, Min Hoan Moon, Kyung Chul Moon, Sang Youn Kim

**Affiliations:** 1 Department of Radiology, Ewha Womans University College of Medicine, Seoul, Republic of Korea; 2 Department of Radiology, Seoul National University Hospital, Seoul, Republic of Korea; 3 Department of Radiology, Seoul National University College of Medicine, Seoul, Republic of Korea; 4 Department of Radiology, SMG-SNU Boramae Medical Center, Seoul National University College of Medicine, Seoul, Republic of Korea; 5 Department of Pathology, Seoul National University Hospital, Seoul, Republic of Korea

**Keywords:** Kidney neoplasms, Papillary renal cell carcinoma, Multidetector computed tomography, Radiomics, Differential diagnosis

## Abstract

**Purpose:**

To assess the computed tomography (CT) findings of papillary renal neoplasm with reverse polarity (PRNRP) and develop a radiomics-based model to distinguish PRNRPs from papillary renal cell carcinomas (PRCCs).

**Materials and methods:**

We analyzed 31 PRNRPs and 68 PRCCs using preoperative kidney CT. We evaluated CT features that could discriminate PRNRPs from PRCCs. A radiomics signature was constructed using features selected through a least absolute shrinkage and selection operator algorithm. A radiomics-based model incorporating a radiomics signature and subjective CT parameters using multivariate logistic regression was developed. The diagnostic performance of the CT parameters, radiomics model, and their combination was evaluated using the area under the curve (AUC).

**Results:**

Most of PRNRPs had a round shape (93.5%), well-defined margin (100%), and persistent enhancement (77.4%). Compared with PRCC, PRNRPs exhibited distinct CT features including small size (16.7 vs. 37.7 mm, *P* < 0.001), heterogeneity (64.5 vs. 32.4%, *P* = 0.004), enhancing dot sign (16.1 vs. 1.5%, *P* = 0.001), and high attenuation in pre-contrast CT (44.2 vs. 35.5 HU, *P* = 0.003). Multivariate analysis revealed smaller mass size (odds ratio [OR]: 0.9; 95% confidence interval [CI] 0.9–1.0, *P* = 0.013), heterogeneity (OR: 8.8; 95% CI 1.9–41.4, *P* = 0.006), and higher attenuation in pre-contrast CT (OR: 1.1; 95% CI 1.0–1.2, *P* = 0.011) as significant independent factors for identifying PRNRPs. The diagnostic performance of the combination model was excellent (AUC: 0.923).

**Conclusion:**

Smaller tumor size, heterogeneity, and higher attenuation in pre-contrast CT were more closely associated with PRNRPs than with PRCCs. Though the retrospective design, small sample size, and single-center data of this study may affect the generalizability of the findings, combining subjective CT features with a radiomics model is beneficial for distinguishing PRNRPs from PRCCs.

## Introduction

Papillary renal cell carcinoma (PRCC) is the second most common type of renal cell carcinoma [1]. PRCC has traditionally been classified into types 1 (PRCC1) and 2 (PRCC2) based on its histology and prognosis [2]. However, many tumors with papillary architecture that were previously categorized as PRCC1 or PRCC2 are now considered distinct and separate entities [3]. As the understanding of PRCC evolves and includes independent tumors with specific molecular or clinical features, the perception of traditional PRCC has changed. According to the fifth edition of the World Health Organization (WHO) classification in 2022, subdividing PRCC into PRCC1 and 2 is no longer recommended. PRCC1 is currently considered ‘classic PRCC’, while renal tumors previously labeled PRCC2 exhibit significantly variable morphologies and clinical characteristics.

Papillary renal neoplasm with reverse polarity (PRNRP) is one of the entities newly recognized as a distinct tumor subtype with unique morphological patterns [4]. Notably, several pathological studies have been conducted on PRNRP [5–7]. Histologically, PRNRP is characterized by a papillary neoplasm with low-grade nuclear features, an inverted nuclear location (linear nuclear arrangement apart from the base), and abundant eosinophilic cytoplasm. PRNRP is constantly positive for GATA3 and negative for vimentin, and has recurrent KRAS mutations. However, to our knowledge, the imaging findings of PRNRP have not yet been reported. PRNRP is classified as a malignant tumor; however, it typically exhibits indolent biological behavior, with most tumors being small and no cases of disease-specific death reported during the follow-up period [5, 8]. Since clinical features of PRNRP are similar to benign tumors and have no metastasis or recurrence, accurately distinguishing PRNRP from PRCC is essential for proper clinical management.

Radiomics provides quantitative image information that exceeds the visual interpretation ability of radiologists [9]. It has been widely used to differentiate various renal tumor types [10, 11]. Combining subjective image features with a radiomics-based model may help differentiate PRNRPs from PRCCs.

Therefore, this study aimed to analyze subjective computed tomography (CT) findings of PRNRPs and developed a radiomics-based model combined with CT parameters to differentiate between PRNRPs and PRCCs.

## Materials and methods

### Study cohort

**Fig. 1:**
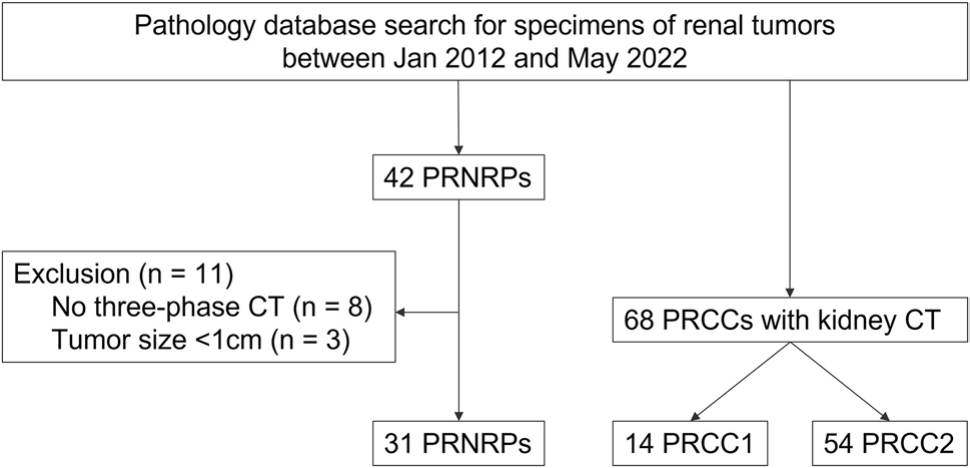
Flowchart of patient selection. *PRNRP* papillary renal neoplasm with reverse polarity, *PRCC* papillary renal cell carcinoma, *PRCC1* type 1 Papillary renal cell carcinoma, *PRCC2* type 2 Papillary renal cell carcinoma

This retrospective case–control study was approved by the Institutional Review Board, which waived the need to obtain informed consent from the patients. We searched the pathological database of our institution for surgical specimens diagnosed as papillary renal tumors with oncocytic cytoplasm between January 2012 and May 2022. Because PRNRPs are a recently defined emerging entity, an experienced pathologist with extensive subspecialty training in genitourinary pathology reviewed all pathological slides of papillary tumors with oncocytic cytoplasm and diagnosed 42 patients with PRNRP. We excluded eight patients who did not undergo a three-phase kidney protocol CT, including pre-contrast, corticomedullary phase (CMP), and excretory phase (EP), and three with tiny tumors < 1 cm in size because tiny tumors were inadequate for subjective image analysis. Finally, 31 patients were included in this study (Fig. 1).

To gather a comparison group, twice the size of the PRNRP cases, we selected 68 consecutive patients diagnosed with PRCC who underwent an appropriate kidney protocol CT between January 2018 and May 2022. This group included 14 patients with PRCC1 and 54 with PRCC2.

### Subjective image analysis

CT scans were acquired using 3 CT scanners (SOMATOM Force; Siemens Healthineers, IQon; Philips Healthcare, Revolution Frontier; GE Healthcare) with 3 mm slick thickness. CMP images were obtained 17 s after the descending aorta reached a threshold of 150 HU. For EP imaging, images were acquired 3 min after IV contrast injection. Two fellowship-trained radiologists who specialized in genitourinary imaging for 10 and 5 years, respectively, independently reviewed the CT scans of PRNRPs and PRCCs. A third radiologist, possessing 20 years of expertise in genitourinary imaging, resolved the disagreement. The readers were blinded to the patient information and histopathological results. We evaluated the following characteristics: maximal diameter of the tumor; tumor laterality (right or left); exophytic/endophytic properties (≥ 50% exophytic, < 50% exophytic, or complete endophytic); tumor shape (round or lobulated); long-to-short axis ratio (LSR); tumor heterogeneity (homogeneous or heterogeneous); the presence of enhancing dot sign; the presence of calcification; grade of tumor necrosis (< 33%, 34–66%, or ≥ 67%); tumor margin (well-defined or infiltrative); enhancement pattern (wash-in and wash-out or persistent enhancement); attenuation in pre-contrast scan; tumor-to-cortex enhancement ratio (TCR) in the CMP and EP; renal vein invasion; lymph node metastasis; and distant metastasis.

The LSR was calculated as the ratio of the longest to shortest axes of the tumors. Tumors were described as homogeneous when > 90% of the area appeared to have similar attenuation on visual inspection in the CMP; otherwise, they were considered heterogeneous. An enhancing dot sign was defined as a tiny dot-like enhancement within the tumor in the CMP. The tumor margin was classified as well-defined when the tumor edge was clear; otherwise, they were considered irregular. The enhancement pattern was considered wash-in and wash-out when the attenuation of the solid portion of the tumor decreased by at least 20 Hounsfield units (HU) in the EP compared with that in the CMP; otherwise, it was considered persistent enhancement [11]. In all three phases, we drew regions of interest (ROIs) that were as large as possible in the solid portion of the tumor, avoiding necrotic or hemorrhagic portions. In addition, we placed ROIs in the renal cortex in the CMP and EP. The TCR was calculated by dividing the HU of the tumor by that of the cortex in the CMP and EP.

### Tumor segmentation and feature extraction

The lesions were manually segmented on all slices using commercially available software (MEDIP, Medical IP). Two radiologists independently delineated the tumors semi-automatically using intensity-based thresholding and a region-growing function in the CMP images. The copied masks were pasted onto the pre-contrast images, followed by further manual refinement.

We used a free and open-source software, 3D Slicer (http://www.slicer.org), for feature extraction. A total of 107 radiomics features were automatically extracted in each phase using the 3D Slicer pyradiomics module. Image resampling was performed at a spatial resolution of 1 × 1 × 1 mm^3^ using linear interpolation and discretized with a bin width of 25 HU. In addition, to minimize CT intensity changes and obtain more stable radiomics features, we normalized the image intensity using the following formula [12]:

$$f\left(x\right)= \frac{s (x- {\upmu }_{x})}{{\upsigma }_{x}}$$

where *x* represents the original intensity, *f*(*x*) represents the normalized intensity, μ indicates the average value, σ refers to variance, and s is an optional scaling ratio, which has been set to 1 by default.

A total of 214 radiomics features were extracted, including the following categories: first-order statistics, shape features, gray-level co-occurrence matrices (GLCMs), gray-level run-length matrices, gray-level size zone matrices, neighboring gray tone difference matrices, and gray-level dependence matrices.

### Feature selection and radiomics model development

**Fig. 2:**
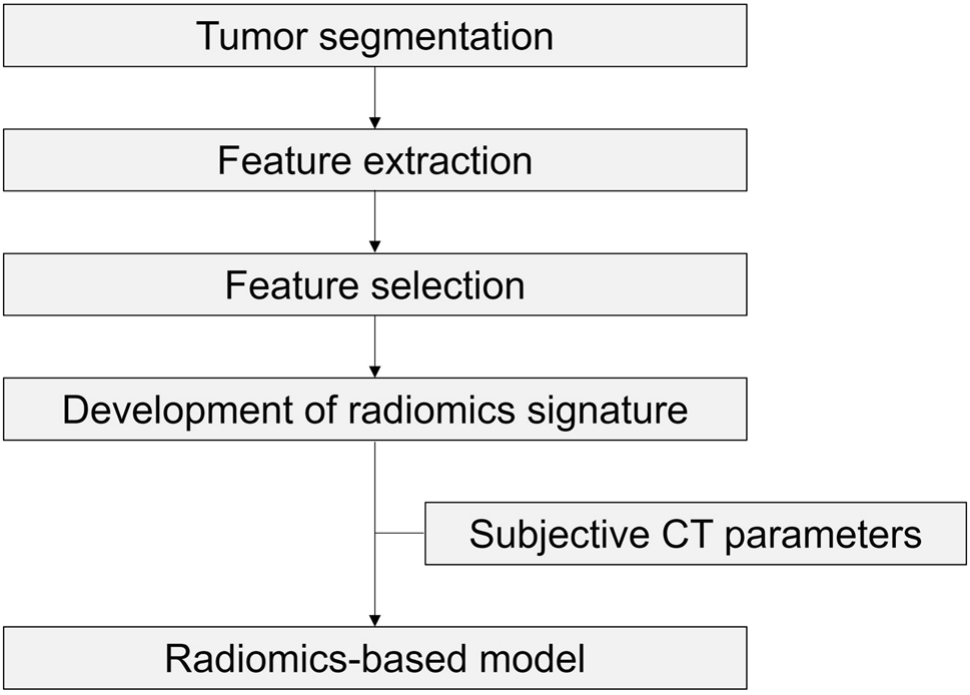
Radiomics workflow. Tumors were manually segmented in CT images by two radiologists. Radiomics features were extracted, and the reliable features were selected using the intra-class correlation coefficient and the least absolute shrinkage and selection operator (LASSO) logistic regression algorithm. A radiomics signature for distinguishing PRNRP was then developed. Subsequently, a radiomics-based model was created by incorporating the significant subjective CT parameters

We selected reliable radiomics features with an intra-class correlation coefficient (ICC) ≥ 0.75 in the interobserver study. The least absolute shrinkage and selection operator (LASSO) logistic regression algorithm was applied to select features from those determined to be reliable. Ten-fold cross-validation was applied to tune the regularization parameters. A radiomics signature for distinguishing PRNRP was developed using a multivariate logistic regression model with selected features. The radiomics signature and significant subjective CT parameters were incorporated into a radiomics-based model to diagnose PRNRP. The overall radiomics workflow is visualized in Fig. 2.

### Statistical analysis

We used Fisher’s exact and Mann–Whitney *U* tests for categorical and continuous variables, respectively, in group comparisons. We compared PRNRP with the entire PRCC group, and then we compared PRNRP with the PRCC1 and PRCC2 subgroups separately. To determine the significant factors for identifying PRNRPs, we conducted a univariate logistic regression analysis of the subjective CT parameters and radiomics signature. Significant parameters with *P* < 0.05 were used for multivariate analysis to develop the combined model of subjective CT parameters and the radiomics model. Interobserver agreement was evaluated using Cohen’s kappa coefficient and ICC for categorical and continuous variables, respectively. The kappa values were defined as follows: *κ* < 0, less than chance agreement; 0.01–0.20, slight agreement; 0.21–0.40, fair agreement; 0.41–0.60, moderate agreement; 0.61–0.80, substantial agreement; and 0.81–0.99, almost perfect agreement. For ICC, values ≥ 0.75 were considered reproducible. Receiver operating characteristic curve analyses were performed to evaluate the performance of the prediction model. We compared the AUC of the subjective CT parameters, radiomics model, and combined model. Optimal cutoff values for the significant factors were obtained using the Youden index J. All statistical analyses were performed using SPSS (version 25.0; IBM, Armonk, NY), and MedCalc Statistical Software (version 22.009; MedCalc Software Ltd, Osted, Belgium). Statistical significance was set at *P* < 0.05.

## Results

### Patients’ baseline characteristics

Patients’ baseline characteristics are summarized in Tables 1. Patients with PRNRP were significantly younger than those with PRCC (58.9 ± 11.6 vs. 64.0 ± 10.8 years, *P* = 0.037). When classifying PRCC types, the mean age of the patients with PRNRP was significantly younger than that of those with PRCC2 (58.9 ± 11.6 vs. 64.7 ± 11.0 years, *P* = 0.024). In contrast, there was no statistically significant difference with PRCC1 (*P* = 0.529). The male predominance was significantly higher in the PRCC group than in the PRNRP group (83.8% vs. 58.1%, *P* = 0.010). No patients in the PRNRP group presented with renal vein invasion, lymph node invasion, or distant metastasis.

**Table 1:** Clinical and radiological characteristics of patients according to the tumor groups

Characteristics	PRNRP (*n* = 31)	PRCC (*n* = 68)	PRNRP vs. PRCC *P* value^a^	PRCC 1 (*n* = 14)	PRNRP vs. PRCC1 *P* value^a^	PRCC 2 (*n* = 54)	PRNRP vs. PRCC2 *P* value^a^
Age (years)	58.9 ± 11.6	64.0 ± 10.8	0.037	61.2 ± 10.1	0.529	64.7 ± 11.0	0.024
Sex			0.010		0.094		0.019
Men	18 (58.1)	57 (83.8)		12 (85.7)		45 (83.3)	
Women	13 (41.9)	11 (16.2)		2 (14.3)		9 (16.7)	
Tumor size (mm)	16.7 ± 8.7	37.7 ± 22.3	< 0.001	36.1 ± 20.9	0.004	38.1 ± 22.9	< 0.001
Laterality			0.189		0.326		0.177
Right	10 (32.3)	33 (48.5)		7 (50.0)		26 (48.1)	
Left	21 (67.7)	35 (51.5)		7 (50.0)		28 (51.9)	
Exophytic/endophytic			0.401		0.607		0.366
≥ 50% exophytic	17 (54.8)	46 (67.6)		10 (71.4)		36 (66.7)	
< 50% exophytic	9 (29.0)	16 (23.5)		2 (14.3)		14 (25.9)	
Complete endophytic	5 (16.1)	6 (8.8)		2 (14.3)		4 (7.4)	
Shape			0.136		0.578		0.120
Round	29 (93.5)	55 (80.9)		12 (85.7)		43 (79.6)	
Lobulated	2 (6.5)	13 (19.1)		2 (14.3)		11 (20.4)	
LSR (%)	1.21 ± 0.16	1.28 ± 0.20	0.089	1.29 ± 0.18	0.147	1.28 ± 0.20	0.098
Tumor heterogeneity			0.004		< 0.001		0.026
Homogeneous	11 (35.5)	46 (67.6)		13 (92.9)		33 (61.1)	
Heterogeneous	20 (64.5)	22 (32.4)		1 (7.1)		21 (38.9)	
Enhancing dot sign	5 (16.1)	1 (1.5)	0.011	0 (0)	0.305	1 (1.9)	0.020
Calcification	0 (0)	6 (8.8)	0.173	1 (7.1)	0.311	5 (9.3)	0.153
Grade of necrosis			0.776		1.000		0.646
< 33%	28 (90.3)	58 (85.3)		14 (100)		44 (81.5)	
33–66%	2 (6.5)	6 (8.8)		0 (0)		6 (11.1)	
≥ 67%	1 (3.2)	4 (5.9)		0 (0)		4 (7.4)	
Margin			0.173				0.082
Well-defined	31 (100)	62 (91.2)		14 (100)		48 (88.9)	
Infiltrative	0 (0)	6 (8.8)		0 (0)		6 (11.1)	
Enhancement pattern			0.226		0.402		0.362
Wash-in and wash-out	7 (22.6)	8 (11.8)		1 (7.1)		7 (13.0)	
Persistent enhancement	24 (77.4)	60 (88.2)		13 (92.9)		47 (87.0)	
HU in pre-contrast	44.2 ± 14.2	35.5 ± 7.3	0.003	34.7 ± 6.9	0.004	35.7 ± 7.4	0.004
TCR in CMP	0.46 ± 0.15	0.36 ± 0.17	0.010	0.30 ± 0.13	0.002	0.38 ± 0.18	0.043
TCR in EP	0.50 ± 0.13	0.42 ± 0.14	0.003	0.41 ± 0.14	0.026	0.42 ± 0.14	0.006

Categorical variables are expressed as numbers with percentages in parentheses, whereas continuous variables are expressed as mean ± standard deviation

*PRNRP* papillary renal neoplasm with reverse polarity, *PRCC* papillary renal cell carcinoma, *PRCC 1* type 1 papillary renal cell carcinoma, *PRCC 2* type 2 papillary renal cell carcinoma, *LSR* long-to-short axis ratio, *HU* Hounsfield unit, *TCR* tumor-to-cortex enhancement ratio, *CMP* corticomedullary phase, *EP* excretory phase

^*^Continuous data were assessed using the Mann–Whitney U test, whereas categorical data were assessed using Fisher’s exact test

### Comparison of CT findings between PRNRPs and PRCCs

The mean tumor size was significantly smaller in the PRNRP group than in the PRCC group (16.7 ± 8.7 vs. 37.7 ± 22.3 mm, *P* < 0.001). Furthermore, most PRNRPs were round-shaped (93.5%) and had well-defined margins (100%). Tumor necrosis and calcification were rare. Tumors mostly exhibited weak enhancement compared with the renal cortex (TCR: 0.46 and 0.50 in the CMP and EP, respectively) and a persistent enhancement pattern (77.4%). PRNRPs showed a more heterogeneous enhancement than PRCCs (64.5% vs. 32.4%, *P* = 0.004). PRNRPs showed more frequent enhancing dot signs than PRCCs (16.1% vs. 1.5%, *P* = 0.011). PRNRPs tended to have higher attenuation in pre-contrast images (44.2 ± 14.2 vs. 35.5 ± 7.3 HU, *P* = 0.003) and higher TCR in the CMP (0.46 ± 0.15 vs. 0.36 ± 0.17, *P* = 0.010) and EP images (0.50 ± 0.13 vs. 0.42 ± 0.14, *P* = 0.003). The results of the subjective CT analysis according to the tumor groups are presented in Table 1. PRNRPs showed a significantly smaller tumor size, higher heterogeneity, higher pre-contrast CT number, and higher TCR in the CMP and EP than PRCC1 and 2 (all *P* < 0.05). The enhancing dot sign was rarely observed in all tumors (PRNRP, 5%; PRCC1, 0%; PRCC2, 1%); however, statistically significant differences were observed between PRNRP and PRCC2 (*P* = 0.020).

### Radiomics feature extraction and development of the radiomics prediction model

Among the 214 radiomics features extracted, we excluded 48 from the CMP and 64 from the pre-contrast image with unacceptable interobserver agreement on image segmentation (ICC < 0.75). We used 102 features (59 from the CMP and 43 from the pre-contrast image) to develop the radiomics model. Finally, the two most relevant features from the CMP (one three-dimensional shape-based and one GLCM) and one from the pre-contrast image (GLCM) were selected and used to develop the radiomics signature (Table 2).

**Table 2:** Radiomics features finally selected to develop a radiomics signature

Image Contrast	Feature Classification	Feature Name	Outcome	
			PRNRP	PRCC
CMP	Shape-based (3D)	Surface Area to Volume Ratio	0.53 ± 0.20	0.31 ± 0.17
Pre-contrast	GLCM	IMC1	–0.12 ± 0.07	–0.07 ± 0.03
CMP	GLCM	IMC1	–0.16 ± 0.07	–0.11 ± 0.04

*PRNRP* papillary renal neoplasm with reverse polarity, *PRCC* papillary renal cell carcinoma, *CMP* corticomedullary phase, *GLCM* gray-level co-occurrence Matrix, *IMC1* Informational Measure of Correlation 1

### Interobserver agreement

The interobserver agreement between two radiologists for tumor heterogeneity was fair (*κ* = 0.51). Two readers showed moderate to almost perfect agreement for other categorial variables (*κ* = 0.63–1.0), and reliable ICCs (0.85–0.93) were observed for continuous variables (Table 3).

**Table 3:** Interobserver agreements of CT parameters in subjective image analysis in the study population between two readers

	Agreement^a^	Standard error	95% CI
Exophytic/endophytic	0.64	0.06	0.51–0.77
Shape	0.81	0.13	0.57–1.0
Tumor heterogeneity	0.51	0.08	0.35–0.69
Enhancing dot sign	0.63	0.15	0.33–0.92
Calcification	1.0	0	1.0–1.0
Grade of necrosis	0.88	0.12	0.65–1.0
Margin	0.73	0.18	0.38–1.0
Enhancement pattern	0.80	0.08	0.64–0.96
HU in pre-contrast	0.92	–	0.62–0.97
TCR in CMP	0.93	–	0.89–0.96
TCR in EP	0.85	–	0.75–0.91

*CI* confidence interval, *HU* Hounsfield unit, *CMP* corticomedullary phase; *EP*, excretory phase

^a^Agreements regarding categorical and continuous variables were assessed with Cohen’s κ statistics and intra-class correlation coefficients, respectively

### Univariate and multivariate analyses of CT parameters for identifying PRNRPs

**Fig. 3:**
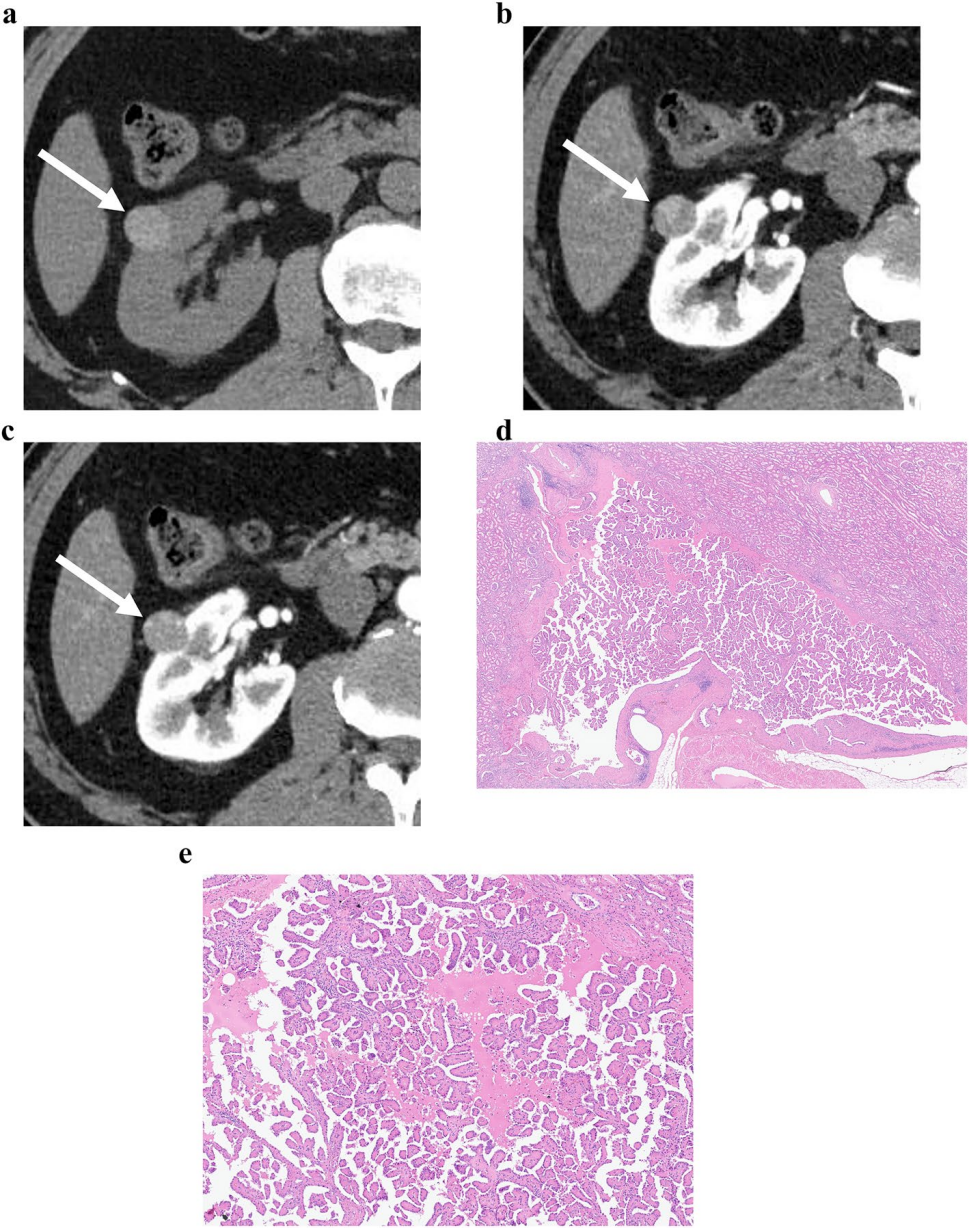
CT and histologic images of a 64-year-old man with papillary renal neoplasm with reverse polarity. **a** Pre-contrast CT image showed a 19-mm tumor with high attenuation (80 HU) in the right kidney interpolar area. The tumor demonstrated heterogeneous enhancement (**b**) and enhancing dot sign (**c**) in the corticomedullary phase. **d** A low magnification view showed thin papillary architecture with delicate fibrovascular core. **e** In a high magnification view, tumor cells showed eosinophilic, finely granular cytoplasm and the nuclei were low grade and apically located

**Fig. 4:**
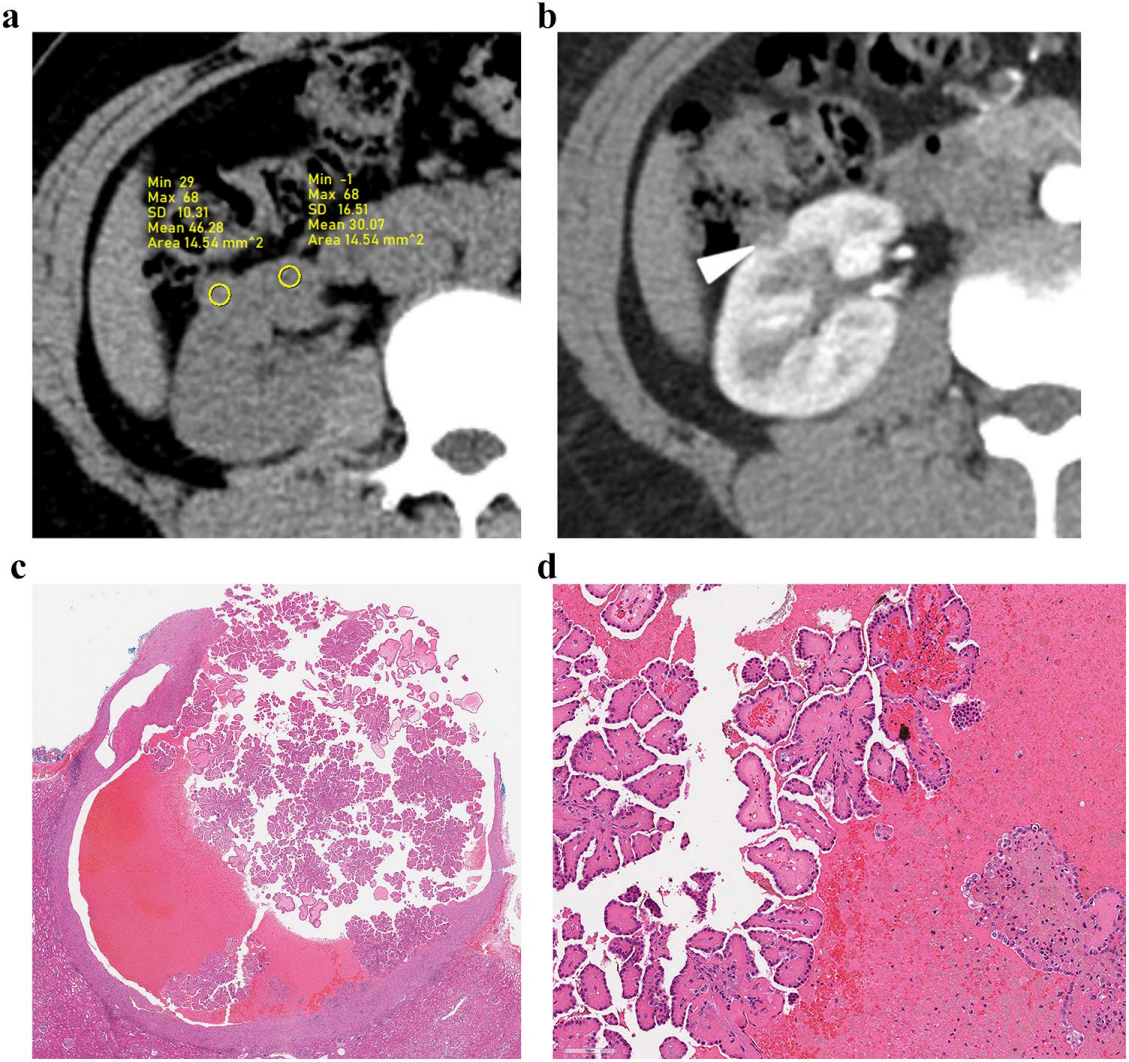
A case of papillary renal neoplasm with reverse polarity with cystic and hemorrhagic change in 47-year-old woman. **a** Pre-contrast CT image showed a 12-mm tumor with high attenuation (46 HU) in the right kidney interpolar area. **b** The tumor demonstrated heterogeneous enhancement and enhancing dot sign (arrowhead) in the corticomedullary phase. **c**, **d** In these histologic images, the tumor demonstrated papillary architecture with hemorrhagic and cystic changes

**Fig. 5:**
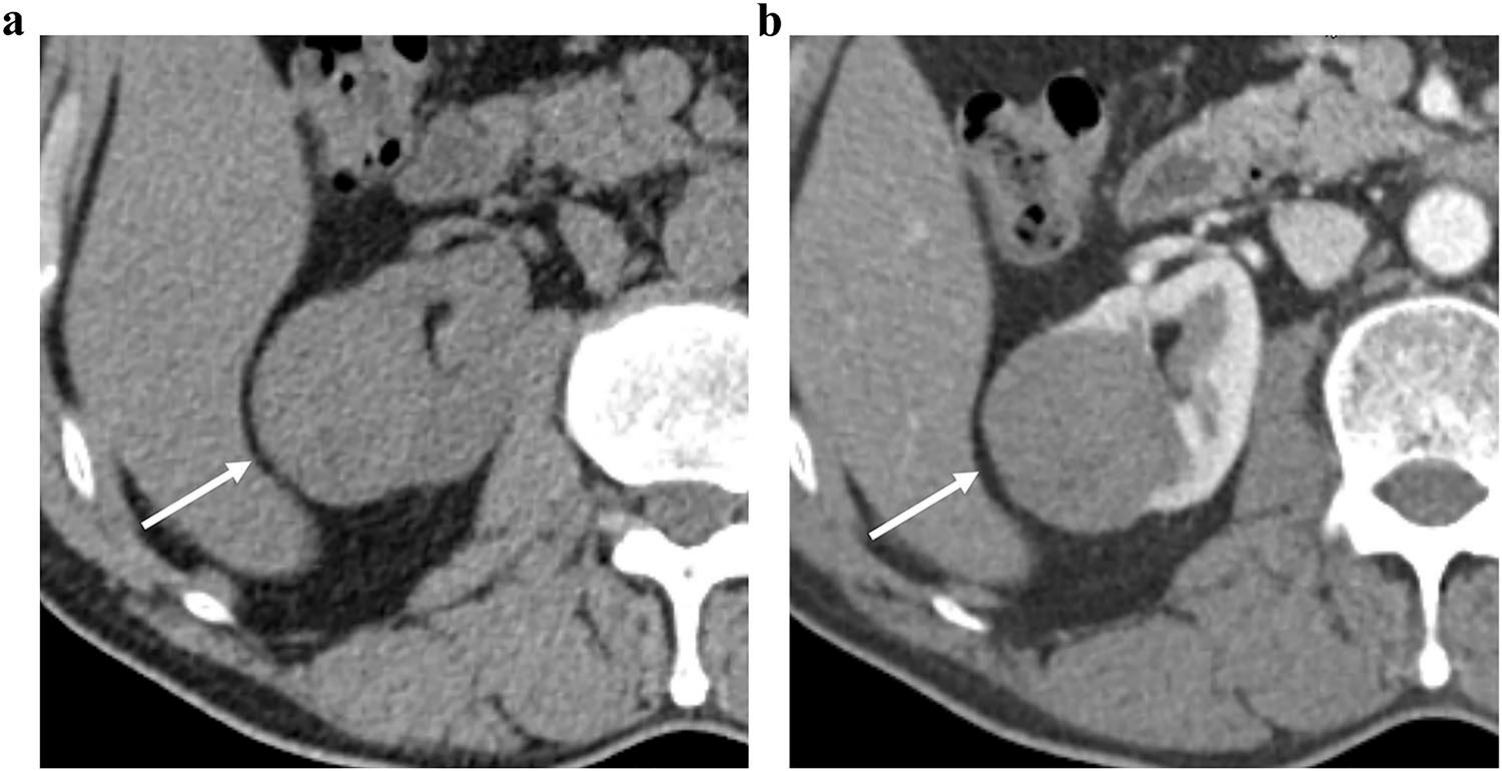
A 66-year-old man with type 1 papillary renal cell carcinoma. **a** The pre-contrast CT image showed a 43-mm tumor with low attenuation (35 HU) in the left kidney lower pole. **b** The tumor showed homogeneous enhancement in the corticomedullary phase

Univariate and multivariate analyses of CT parameters for identifying RPNRPs are presented in Table 4. In the univariate analysis, small tumor size, heterogeneous enhancement, enhancing dot sign, higher attenuation in pre-contrast CT, higher TCR in the CMP and EP, and radiomics signature were significant factors for identifying PRNRPs (*P* < 0.05). In the multivariate logistic analysis, small tumor size (odds ratio [OR]: 0.9; 95% confidence interval: [CI] 0.9–1.0, *P* = 0.013), heterogeneity (OR: 8.8; 95% CI 1.9–41.4, *P* = 0.006), high attenuation on pre-contrast CT (OR: 1.1; 95% CI 1.0–1.2, *P* = 0.011), and radiomics signature (OR: 25.5; 95% CI 1.2–535.8, *P* = 0.037) were significant independent factors for distinguishing PRNRPs from PRCCs (Figs. 3, 4, 5).

**Table 4:** Univariate and multivariate analyses of subjective CT parameters and radiomics features for identifying PRNRP

Parameters	Univariate analysis		Multivariate analysis	
	Odds ratio (95% CI)	*P* value	Odds ratio (95% CI)	*P* value
Tumor size	0.9 (0.8–0.9)	< 0.001	0.9 (0.9–1.0)	0.013
Heterogeneity	3.8 (1.6–9.3)	0.003	8.8 (1.9–41.4)	0.006
Enhancing dot sign	12.9 (1.4–115.6)	0.022	0.5 (0.0–7.8)	0.650
HU in pre-contrast	1.1 (1.0–1.1)	0.006	1.1 (1.0–1.2)	0.011
TCR in CMP	26.1 (2.0–346.5)	0.011	23.8 (0.9–6356.1)	0.266
TCR in EP	140.3 (4.4–4521.4)	0.003	0.4 (0.0–430.5)	0.781
Radiomics signature	199.6 (22.1–1803.6)	< 0.001	25.5 (1.2–535.8)	0.037

*PRNRP* papillary renal neoplasm with reverse polarity, *CI* confidence interval, *HU* Hounsfield unit, *TCR* tumor-to-cortex enhancement ratio, *CMP* corticomedullary phase, *EP* excretory phase

### Diagnostic performance of the radiomics and combined models

**Fig. 6:**
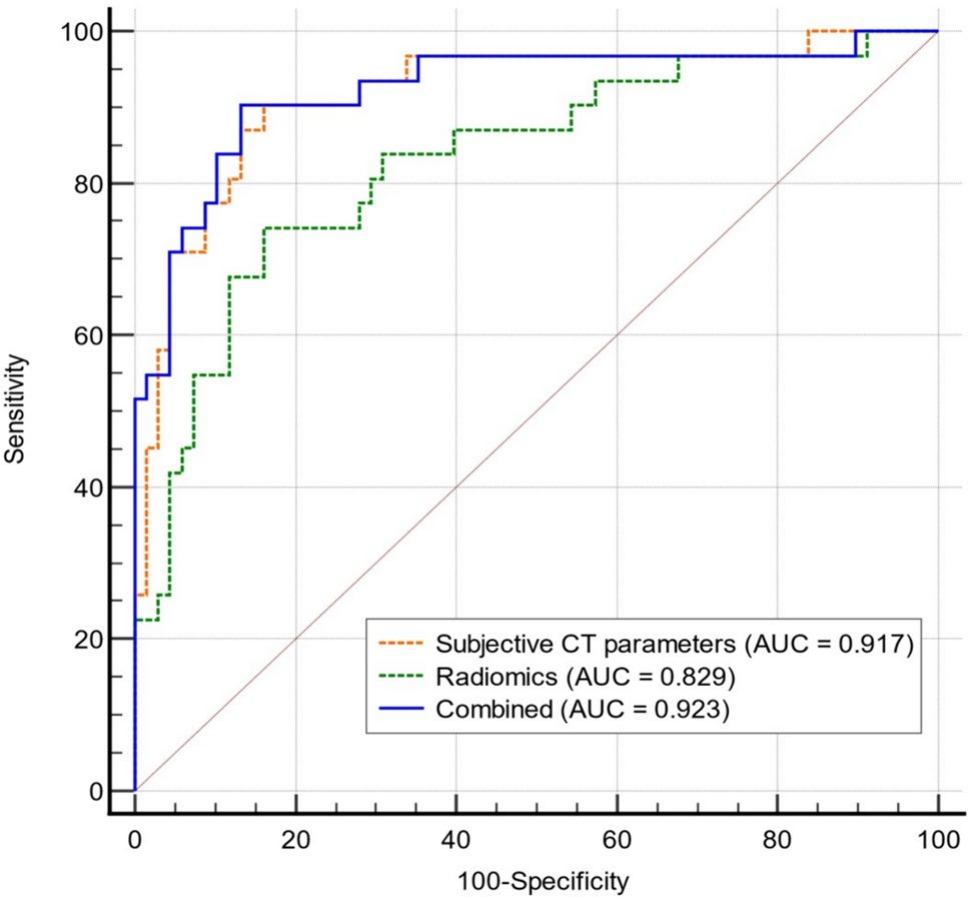
Receiver-operating characteristic curve of the subjective CT parameters, radiomics model, and combined model for differentiating between papillary renal neoplasm with reverse polarity and papillary renal cell carcinoma

The diagnostic performances of the three subjective CT parameters and radiomics signature are summarized in Table 5. The optimal cutoff values for mass size, attenuation in pre-contrast CT, and the radiomics signature were 23 mm, 32 HU, and 0.34, respectively. The AUCs of the subjective CT parameters were 0.92 (95% CI: 0.85–0.96), with a sensitivity of 90.3% and a specificity of 83.8%. The radiomics signature showed an AUC of 0.83 (0.74–0.90), with a sensitivity of 74.2% and a specificity of 83.8%. The combined model showed an AUC of 0.92 (0.85–0.97), with a sensitivity of 90.3% and a specificity of 86.8% (Fig. 6).

**Table 5:** Diagnostic performance of the three significant CT parameters and radiomics signature in identifying PRNRP

Parameters	Sensitivity (%)	Specificity (%)	Accuracy (%)
Mass size (≤ 23 mm)	93.5	64.7	73.7
Heterogeneity	64.5	67.6	66.7
High attenuation in pre-contrast (> 32 HU)	90.3	39.7	55.6
Radiomics signature (> 0.34)	74.2	83.8	80.8

*PRNRP* papillary renal neoplasm with reverse polarity, *HU* Hounsfield unit

## Discussion

PRNRP is a distinct subtype of papillary renal tumors and was newly named in 2019. It differs from PRCC1 and 2 in molecular, clinical, pathological, and immunohistochemical features [13]. Notably, most PRNRPs are diagnosed incidentally and staged as pT1. Due to its indolent clinical behavior, differentiating PRNRP from other papillary renal tumors is important. In the present study, we identified CT findings of small size, heterogeneity, and high attenuation in pre-contrast CT of PRNRPs. To our knowledge, the present study is the first imaging study to investigate the CT features of PRNRP with a sufficient number of cases.

PRNRP has been indicated as various terms, including oncocytic PRCC [14], adult papillary renal tumor with oncocytic cells [15], PRCC with oncocytic cells and non-overlapping low-grade nuclei [16], and oncocytic PRCC with inverted nuclear pattern [17]. Oncocytic PRCCs are tumors with voluminous, granular, eosinophilic cytoplasm, regardless of nuclear morphology [18, 19]. PRNRP is a subtype of oncocytic PRCC; however, it has distinct, apically located nuclei with low-grade features. Al-Obaidy et al. proposed the term “PRNRP” in 2019, which has been accepted as appropriate [13]. A case report showed the CT features of oncocytic PRCCs, including six tumors [20]. The researchers suggested that oncocytic PRCC has CT features similar to those of PRCC1 rather than those of PRCC2. However, they may have included various types of oncocytic PRCC and not just PRNRP.

There are only two recently published case reports on the CT findings of three PRNRPs [21, 22]. One study reported that a tumor had isodensity to the renal parenchyma in pre-contrast CT and an inhomogeneous enhancing pattern. The other study reported two highly attenuating masses in a pre-contrast image without obvious enhancement. The high attenuation of PRNRPs in pre-contrast CT and their inhomogeneous enhancement pattern corresponded to the features observed in the present study. Both features are independent factors that distinguish PRNRP from PRCC. Furthermore, these features were more frequently observed in the PRNRP group than in the PRCC1 and 2 groups. We suggested a specific cutoff value of 32 HU for attenuation in the pre-contrast image.

The high attenuation observed in pre-contrast CT can also be seen in fat-poor angiomyolipoma (FP-AML), which is known to be hyperattenuating relative to renal parenchyma due to its smooth muscle component [23]. Differentiating PRNRP from FP-AML can be challenging, particularly since FP-AML typically has a small size, with a mean diameter of approximately 3 cm. However, a key distinguishing feature is their homogeneous enhancement pattern of FP-AML, which contrasts with the heterogeneous enhancement seen in PRNRP. This characteristic can aid in differentiating between these two entities.

The reasons for the higher attenuation in pre-contrast images and the more heterogeneous enhancement pattern of PRNPR compared with PRCC remain unclear. A recent pathological study revealed previously unknown cystic characteristics of PRNRPs. The study reported that the cystic portions within tumors are often filled with proteinaceous fluid or blood [24]. Another study reported a significantly higher cytoplasmic hemosiderin deposition in PRNRP than in PRCC2 [8]. These findings may explain the higher attenuation observed in the pre-contrast images of the PRNRPs (Fig. 4).

Notably, the radiomics features ultimately selected for the radiomics-based model supported the subjective CT features. The values of informational measure of correlation (IMC) 1 of the GLCM features from both the pre-contrast and CMP were lower in the PRNRP group than in the PRCC group, potentially indicating the inherent heterogeneity of PRNRPs. IMC 1 quantifies the complexity of the texture. In addition, among the three-dimensional shape-based features, the surface-area-to-volume ratio derived from the CMP was higher in the PRNRP group than in the PRCC group. This elevated value indicates a less compact and non-spherical shape. This may reflect the softer nature of PRNRP, suggesting its benign nature.

The smaller size of PRNRP compared with PRCC is a well-demonstrated characteristic [5, 8, 13]. In the present study, the average tumor size was significantly smaller in the PRNRP group (16.7 mm) than in the PRCC1 (36.1 mm) and PRCC2 (38.1 mm) groups. A suggested quantitative criterion for distinguishing PRNRP was a size threshold of ≤ 23 mm. Previous studies have established that tumors < 20 mm in size exhibit either a very low or completely absent risk of metastasis [25, 26]. This finding aligns with the characterization of papillary adenoma, the benign counterpart of PRCC, wherein papillary adenoma is classified as a benign tumor rather than a carcinoma based on tumor size [27]. According to the 2016 WHO classification, papillary adenoma is defined as a non-encapsulated papillary and tubulopapillary mass < 15 mm in size that exhibits low-grade nuclear features. Notably, PRNPR has also not shown any instances of metastasis or recurrence [7, 13, 28, 29]. The clinical characteristics of PRNRP appear to be more similar to those of papillary adenomas than those of PRCC. Therefore, it is conceivable that PRNRP may be reclassified as a benign entity in future, similar to papillary adenomas. While existing literature lacks direct comparisons of treatment strategies between PRNRP and PRCC, our findings underscore the potential for revising treatment approaches pending further validation of PRNRP’s benign nature. As accumulating evidence supports the benignity of PRNRP, the paradigm of its management may shift, potentially leading to reclassification as a benign tumor and a decrease in surgical interventions.

This study had several limitations. First, there may have been a selection bias owing to the study’s retrospective nature. Being a single-center investigation with a limited sample size, there is a possibility of inherent biases that could affect the generalizability of our findings. Nonetheless, it is crucial to acknowledge the rarity of PRNRP, which limits the feasibility of larger multi-center studies. Second, the CT images were obtained using various scanners from different vendors. Heterogeneous CT parameters may have influenced radiomics analysis. To mitigate this issue, we employed several processes, including voxel size resampling and CT number normalization. Despite these efforts, it is important to acknowledge the potential influence of imaging variability on our results, and further validation on larger multi-center cohorts with standardized imaging protocols would be beneficial to corroborate our findings and enhance the robustness of our conclusions.

In conclusion, this study thoroughly examined the distinctive CT characteristics of PRNRP and compared them with those of PRCC. The diagnostic performance of the combined model of the radiomics model and CT features was excellent. While there is limited research comparing the treatment strategies for PRNRP and PRCC, the potential to revise treatment options becomes evident if further studies validate the benignity of PRNRP. Consequently, accurate preoperative diagnosis of PRNRP is important for guiding treatment decisions and predicting disease prognosis. We advocate for future research endeavors, particularly larger multi-center studies, to validate our results and pave the way for optimized treatment algorithms tailored to the unique characteristics of PRNRP.
